# Taking Action on Air Pollution Control in the Beijing-Tianjin-Hebei (BTH) Region: Progress, Challenges and Opportunities

**DOI:** 10.3390/ijerph15020306

**Published:** 2018-02-09

**Authors:** Li Wang, Fengying Zhang, Eva Pilot, Jie Yu, Chengjing Nie, Jennifer Holdaway, Linsheng Yang, Yonghua Li, Wuyi Wang, Sotiris Vardoulakis, Thomas Krafft

**Affiliations:** 1 Key Laboratory of Land Surface Pattern and Simulation, Institute of Geographical Sciences and Natural Resources Research, Chinese Academy of Sciences, Beijing 100101, China; wangli@igsnrr.ac.cn (L.W.); yangls@igsnrr.ac.cn (L.Y.); liyh@igsnrr.ac.cn (Y.L.); wangwy@igsnrr.ac.cn (W.W.); 2 Faculty of Health, Medicine and Life Sciences, Maastricht University, 6200 MD Maastricht, The Netherlands; zhangfy@cnemc.cn (F.Z.); eva.pilot@maastrichtuniversity.nl (E.P.); j.yu@maastrichtuniversity.nl (J.Y.); 3 Chinese National Environmental Monitoring Center, Beijing 100012, China; 4 School of Public Administration, Hebei University of Economics and Business, Shijiazhuang 050061, China; chengjingnie@163.com; 5 School of Interdisciplinary Area Studies, University of Oxford, Oxford OX2 6LH, UK; jennifer.holdaway@area.ox.ac.uk; 6 Institute of Occupational Medicine, Edinburgh EH14 4AP, UK; sotiris.vardoulakis@iom-world.org

**Keywords:** air pollution, Beijing-Tianjin-Hebei, air pollution prevention and control action plan, semi-structured interview

## Abstract

Due to rapid urbanization, industrialization and motorization, a large number of Chinese cities are affected by heavy air pollution. In order to explore progress, remaining challenges, and sustainability of air pollution control in the Beijing-Tianjin-Hebei (BTH) region after 2013, a mixed method analysis was undertaken. The quantitative analysis comprised an overview of air quality management in the BTH region. Semi-structured expert interviews were conducted with 12 stakeholders from various levels of government and research institutions who played substantial roles either in decision-making or in research and advising on air pollution control in the BTH region. The results indicated that with the stringent air pollution control policies, the air quality in BTH meets the targets of the Air Pollution Prevention and Control Action Plan. However, improvements vary across the region and for different pollutants. Although implementation has been decisive and was at least in parts effectively enforced, significant challenges remained with regard to industrial and traffic emission control, and national air quality limits continued to be significantly exceeded and competing development interests remained mainly unsolved. There were also concerns about the sustainability of the current air pollution control measures especially for industries due to the top-down enforcement, and the associated large burden of social cost including unemployment and social inequity resulting industrial restructuring. Better mechanisms for ensuring cross-sectoral coordination and for improved central-local government communication were suggested. Further suggestions were provided to improve the conceptual design and effective implementation of respective air pollution control strategies in BTH. Our study highlights some of the major hurdles that need to be addressed to succeed with a comprehensive air pollution control management for the Chinese mega-urban agglomerations.

## 1. Introduction

Air pollution in China is a major concern and it is causing a large public health burden and serious economic losses. According to a recent report, exposure to ambient air pollution has contributed to a death toll exceeding 1.6 million in mainland China, with a total economic loss equivalent to 10.9% of GDP in 2013 [1]. Since 2013, fine particulate matter (PM_2.5_, particulate matter with a mean aerodynamic diameter of 2.5 μm or less) exposure has become the fifth leading cause of death in China, and some 900,000 premature deaths a year are attributable to PM_2.5_ exposure [2], with the highest attributable mortality being in Beijing [3].

Geographically widespread and long-lasting extreme air pollution incidents between 2012 and 2013 (totalling around 3 weeks with hazardous pollution levels in December 2012 and January 2013, covering up to one quarter of China’s land area and affecting up to 600 million people) drew mounting attention not only internationally, but also from the Chinese public [4]. As a consequence, the Chinese government launched a series of ambitious laws and policies to prevent the further deterioration of air quality, and intended to initialise an “improvement phase” in air pollution control in China [5]. Together with the commitment to address climate change (The Paris Agreement 2016) [6], and the commitment to reduce air pollution made by the Chinese premier during his opening address to the 2017 National People’s Congress in Beijing, the enforcement of air pollution prevention and control measures appear to be now in full swing at the national level [4].

The Air Pollution Prevention and Control Action Plan (thereafter the Action Plan), the most stringent air pollution plan to date in China, is regarded as a promising strategy to control deterioration and improve air quality. The plan embedded targets on air quality improvement and emission control into government performance and promotion assessment system, which greatly enhances local officials’ incentives to pay attention to the implementation of the air pollution control measures [7,8,9]. The Action Plan set nation-wide sub-goals for the current five years’ period (2012–2017) with a priority on the three Megalopolises, the Yangtze River Delta (YRD), the Pearl River Delta (PRD) and the Beijing-Tianjin-Hebei region (BTH). Among these, BTH was the most stringently targeted region.

Air pollution control in Beijing traces back to 1998, when it was initiated by the “Blue Sky Project” that however, only focused on point source emissions and pollution control. Since 2004 the preparation for the Olympic Games provided an opportunity to introduce integrated regional prevention and control of air pollution, mainly covering Tianjin, Hebei Province, Shanxi Province, Shandong Province and Inner Mongolia. During the Olympic Games in 2008, the integrated regional prevention and control strategy decreased air pollution to the lowest level one month before and during the games [10]. However, just a few years later in 2011, the USA Embassy in Beijing revealed data indicating that the PM_2.5_ concentration in Beijing was “beyond index” on the United States Environmental Protection Agency (US EPA’s) air quality index [11], which raised international and national public concerns about severe air pollution in China, public awareness of air pollution and the need for effective pollution control strategies. Since then, long-term integrated regional prevention and control of air pollution has been placed prominently on the political agenda. With strengthened control, air quality in the other two megalopolises (YRD and PRD) has improved gradually, but air pollution in BTH is still causing major public health concerns, affecting over 100 million people [12]. According to the first seasonal report on the air quality of the 74 major cities in China in 2016, seven out of the top ten cities with the worst air quality were in the BTH region [13]. Therefore, exploring the progress and the challenges of the Action Plan in BTH is crucial to allow further progress, and—even more-importantly, helping to formulate a more strategic plan in the next stage and to meet the Ministry of Environmental Protection of the People’s Republic of China (MEP’s) target on controlling annual PM_2.5_ concentration below National Ambient Air Quality Standards Level II of 35 µg/m^3^ in all cities by 2030 [4], which, according to recent research, can barely be met with the current policies, particularly in the BTH region [14,15,16].

The novelty of this study is to evaluate the air pollution control policies supported by quantitative analysis and then to explore the challenges and the sustainability of the policies based on qualitative method. Industry and traffic emission control policies are the two foci of this study. The objectives of this study are twofold: (1) to explore the progress of the Air Pollution Prevention and Control Plan; (2) and to identify the opportunities, challenges and sustainability of the air pollution control measures. The aims of this study are to provide a better understanding of the obstacles, problems and risks of the air quality control plan in the BTH region, and to make suggestions on what can be further improved in the near future to make air pollution control strategies more effective and sustainable in China.

## 2. Materials and Methods

A combination of qualitative and quantitative methods was used to assess the progress, challenges and opportunities of the air quality Action Plan in the BTH region. First a quantitative analysis of air quality, indicated by concentrations of the key pollutants and the Air Quality Index (AQI), was used to evaluate the progress in improving air quality. Then interviews with experts were used to explore the progress, the opportunities and the barriers during the implementation, and to provide suggestions for further progress.

### 2.1. Study Area and Quantitative Data

**Figure A1:**
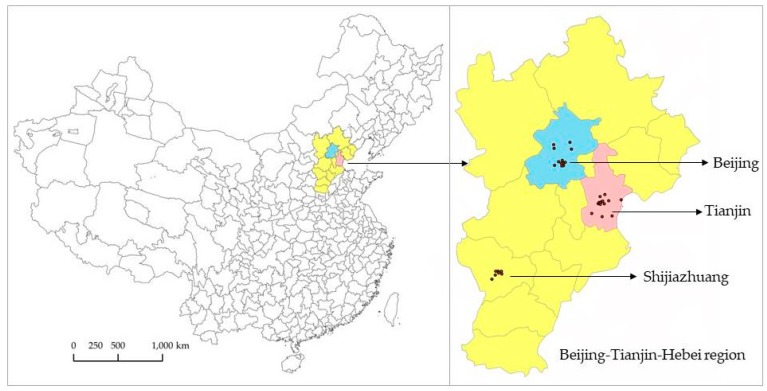
Geo-location of Beijing-Tianjin-Hebei (BTH) region and air quality monitoring stations in Beijing, Tianjin and Shijiazhuang (capital of Hebei Province).

The geo-location of Beijing-Tianjin-Hebei region and the air quality monitoring stations in Beijing, Tianjin and Shijiazhuang (capital of Hebei Province) are shown in Figure A1. Air quality data was obtained from the China National Environmental Monitoring Centre. Air pollution concentration for each city is the mean level from all the monitoring stations at its jurisdiction (Figure A1). The Individual Air Quality Index (IAQI) and Air Quality Index were used to identify the pollution level. The calculation methods for IAQI and AQI, and the classification of AQI were defined by the Technical Regulation on Ambient Air Quality Index (HJ 633-2012) and Ambient Air Quality Standards (GB3095-2012), see Appendix B.

### 2.2. Qualitative Data Collection

Expert interviews were conducted to explore how the control measures of the Action Plan had been implemented and to identify critical challenges that occurred during the implementation within the BTH region with a special focus on industrial restructuring and traffic emission control. As the heavy industrial relocation, mainly from Beijing and Tianjin to Hebei Province, plays a significant role for the industrial restructuring in BTH region, and in particular for the relationship between the regions, we also covered relocation in the interviews. We intended to interview those who played a substantial role in the key government administrations on air pollution control in term of drafting, implementing or supervising the air pollution control measures, and those experts from key institutions who conducted substantial research related to air pollution control and/or provided scientific advice to the governments within the BTH region. After purposive sampling and snowballing, a total of 13 participants were identified of whom 12 responded positively. Eight participants were interviewed face to face, and four provided written statements (interview guidelines see Appendix C). The semi-structured in-depth interviews were conducted from the 18 July to the 30 August 2016. The face to face interview lasted between 45 min to 80 min.

Of the participants interviewed face to face, five were from academic institutions, two from government environmental protection agencies, and one from a non-governmental organization (NGO). The four experts answering via written statements were all from academic institutions. Of the nine academic interviewees, four were from the environmental policy and management field, two were from the field of atmospheric environment research and monitoring, two were from environmental social sciences and one was from environmental health field. Ten participants are from Beijing (BJ), and two were from Hebei Province (HB). As the interview questions were broad, not every interviewee answered all the questions. The interviewees were numbered in the order in which they were interviewed or written statements were received (ID-01, ID-12). After the transcription of the interviews, the interviews were translated from Chinese to English. The interview results and written statement were analysed by thematic analysis in two stages [17,18]. In the first stage we identified four major themes: (1) the interviewees’ interpretation on the air pollution control process; (2) industrial emission control; (3) traffic emission control and (4) air pollution control collaboration. In the second stage, related content was coded to select quotations for each of the four themes.

### 2.3. How the Air Pollution Prevention and Control Action Plan Is Assessed?

There are two assessment aspects for the evaluation of the Action Plan. The first one is the actual improvement of the air quality, quantified as the reduction in particulate matter (PM) concentrations. The second one is the accomplishment of key tasks for air pollution prevention and control, and these tasks include industrial restructuring, clean energy generation, coal and oil quality management, small coal-fired boiler control, industrial emissions (dust and Volatile Organic Compounds—VOCs), municipal dust control, vehicle pollution control, air pollution control investment, building energy-saving and heat metering management, and atmospheric environment management. The two aspects were evaluated separately for BTH, PRD and YRD, and the lower score of these two aspects was the final score. The time frame for the Action Plan is from 2012 to 2017. More detailed information on performance assessment measures of the Action Plan can be found http://www.cleanairchina.org/product/6349.html.

## 3. Results

At the end of 2012 and the beginning of 2013, heavy smog covering the BTH region arose public concern about air pollution. Immediately after the incident, the government launched a series of air quality control strategies including guidelines, laws and other measures. These are listed in Table A2. Among these strategies, the Air Pollution Prevention and Control Action Plan, which included specific measures and assessment methods, acted as the guideline for air pollution control measures. The air pollution reduction targets at provincial or municipal level can be found in Table A3.

### 3.1. Quantitative Result-Air Quality in BTH in the Past 5 Years

**Figure 1:**
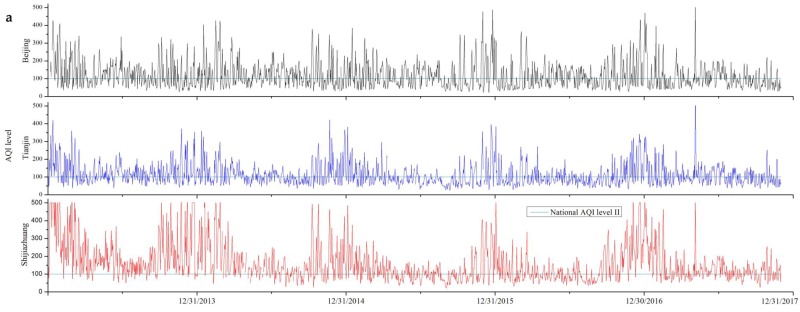

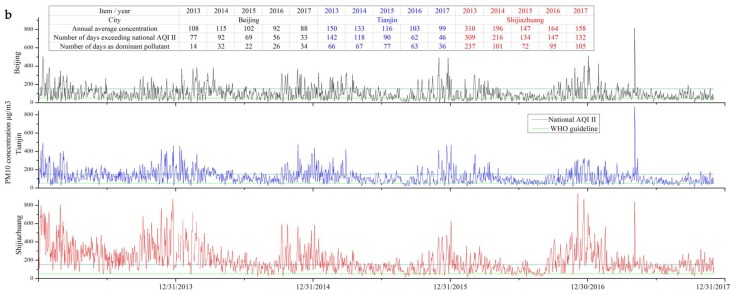

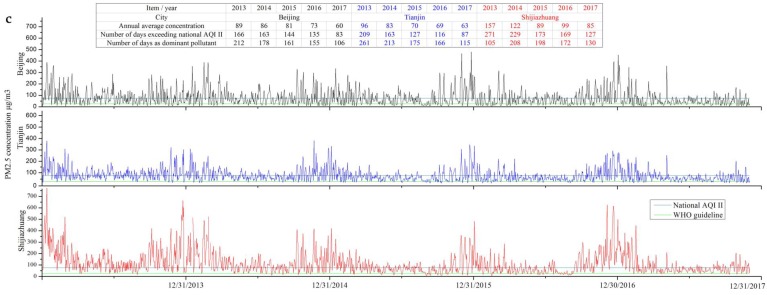

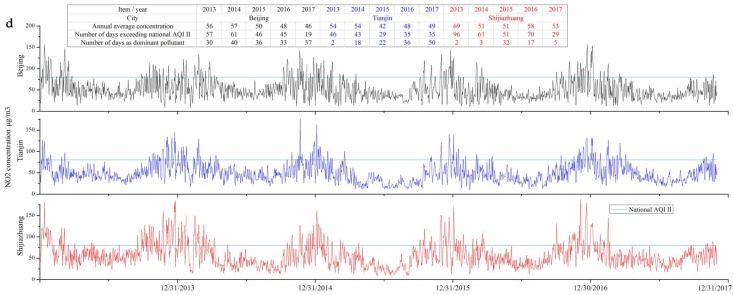

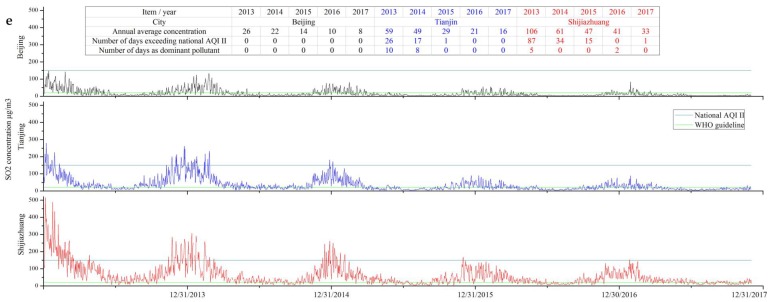

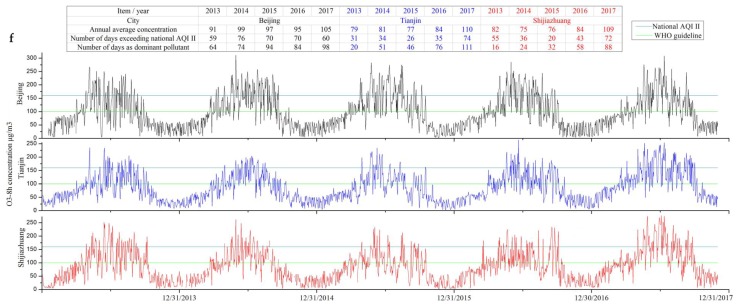
Trends of the daily AQI (**a**); PM_10_ (**b**); PM_2.5_ (**c**); NO_2_ (**d**); SO_2_ (**e**) and 8 h-O_3_ (daily maximum 8 h concentration) (**f**) concentrations in Beijing, Tianjin and Shijiazhuang. According to the Ambient air quality standards (GB3095-2012) and Technical Regulation on Ambient Air Quality Index (HJ 633-2012), below the limit value II, the air quality is good (II) or very good (I); above the limit value II, the air quality is slightly polluted (III), moderately polluted (IV), heavy polluted (V), and sever polluted (VI).

Figure 1 shows the daily trends of Air Quality Index (AQI) and other five major air pollutants (PM_10_, PM_2.5_, O_3_, NO_2_ and SO_2_) in Beijing, Tianjin and Shijiazhuang (the capital city of Hebei Province) from beginning 2013 to November 2017. The annual average concentration, the number of days exceeding AQI level II and WHO guidelines and the number of days as dominant pollutant (see definition in Appendix B) for each pollutant are embed in each figure.

Figure 1 shows clear seasonal change for all the pollutants. The concentrations of PM, NO_x_ and SO_2_ are higher in winter and lower in summer, while O_3_ shows inverse relationship with primary pollutants such as NO_2_, with higher concentration in summer and lower in winter. This is largely attributable to the meteorological conditions and the titration effect between NO_x_ and O_3_ [19,20]. The progress on pollution control has occurred in all three cities but it has been uneven. For PM_10_, all three cities see obvious decline in the past 5 years, particularly for 2016 and 2017. The numbers of days exceeding the national AQI level II are 56 and 33 in 2016 and 2017 for Beijing, 62 and 46 for Tianjin, while 147 and 132 for Shijiazhuang. Similar with PM_10_, PM_2.5_ sees remarkable decrease during the past 5 years. The PM_2.5_ concentrations decrease by 33%, 34% and 45% for Beijing, Tianjin and Shijiazhuang, and Beijing can meet the target of annual average 60 μg/m^3^ from the Action plan. SO_2_ shows a significant decrease in three cities. For Beijing, SO_2_ concentration has met the limit value II since 2013, and for Tianjin and Shijiazhuang, SO_2_ concentration has also met the limit value II since 2016 except for one day. Annual NO_2_ concentration also slightly decreased in the past five years in three cities. O_3_ is the only pollutant showing no steady decline in the past five years, with annual average of the daily maximum 8 h concentration (8 h-O_3_) increasing in Beijing, Tianjin and Shijiazhuang. As a secondary pollutant, the concentration of ozone is strongly but not always positively correlated with the concentration of ozone precursor gases, particularly NOx and VOCs [21,22,23].

Although SO_2_, NO_2_, PM_10_ and PM_2.5_ concentrations in all three cities show decline in the past 5 years, the concentrations of those pollutants are still exceeding AQI level II and are way above the WHO guidelines, particularly in winter time. PM_2.5_ continues to be the dominant pollutant and O_3_ is increasingly becoming the dominant pollutant.

### 3.2. Analysis of the Interviews

In the following we present how the policies that have targeted emissions may have shaped the outcomes above, how the emissions from different sources in the region were controlled, and what kind of challenges and consequences those policies have, based on interviewees’ responses. By means of thematic analysis method, we categorized the interview results into 4 themes “air quality control progress” (Section 3.2.1), “industrial emission control” (Section 3.2.2), “traffic emission control” (Section 3.2.3), and “collaboration on air pollution control” (Section 3.2.4).

#### 3.2.1. Air Quality Control Progress

The air pollution control strategies were introduced in response to concerns about public health and climate change. Although the air quality was still far from reaching the daily limit value II for most regulated pollutants, all the interviewees were optimistic about the air pollution control strategies in BTH, particularly after the Chinese government’s commitment to the Paris Agreement in 2016 [24]. All interviewees indicated that with the implementation of the more stringent air pollution control strategy in the past few years, air quality had improved. Interviewees were asked about all of the three regions that compose BTH were far from reaching the national level II standards (GB 3095-2012; annual mean 70 µg/m^3^ for PM_10_ and 35 µg/m^3^ for PM_2.5_). According to the interviewees, the baseline emissions both from the traffic and the industrial sectors continue being too high because of the rapid economic growth counterbalancing the control measures, accounting for 12.0–25.2% and 11.4–24.6% of the total PM_2.5_ pollution in BTH in 2014 [25], which are the main reasons contributing to the breaching of the national level II standards (GB 3095-2012; annual mean 70 µg/m^3^ for PM_10_ and 35 µg/m^3^ for PM_2.5_). Although vehicle emission standards had been raised to national V or VI level for both gasoline and diesel light duty vehicles in China, which is the same as Euro V or VI for light duty vehicles [26], the total number of vehicles in the BTH region are too huge. Similarly, in the case of heavy industry emission, Hebei had the largest steel industry in China, accounting for a quarter of all steel production nationally. Although the compliance with emission standards has been gradually improving and the government was controlling steel production capacity, heavy emissions from the industrial sectors were still the largest contributor to air pollution in Hebei and Tianjin (ID-03, ID-05) Second, cross-regional transport of pollutants contributed to the high pollution level (ID-04), which can be supported by recent findings indicating that around 28–36% of PM_2.5_ in Beijing was attributable to cross-regional atmospheric transport [27]. Thirdly, the specific climate and topography of Beijing and its surrounding areas were also influencing the dispersion of air pollutants, contributing to high pollution episode [28], also (ID-02). Fourth, even with the stringent control measures, many industrial and vehicle emissions were still violating the emission standards.

#### 3.2.2. Industrial Emission Control

**Figure 2:**
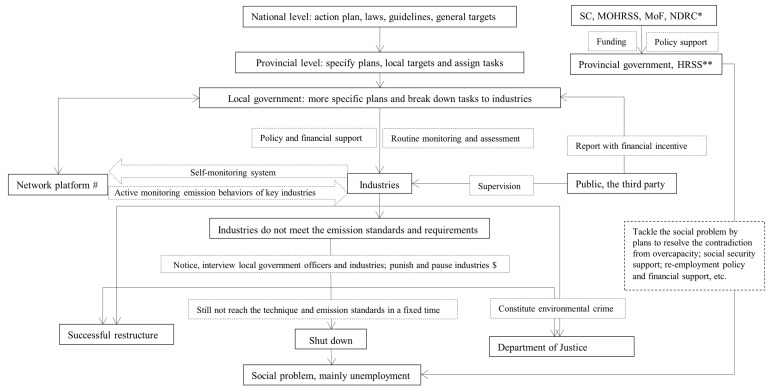
The implementation process of industrial restructuring in Hebei Province. This figure is drawn based on the interview. Solid line indicates institutions or the endpoints of the actions; Dotted line indicates the action process. * SC, State council; MOHRSS, Ministry of Human Resources and Social Security; MoF, Ministry of Finance; NDRC, National Development and Reform Commission. ** HRSS, Department of Human Resources and Social Security. # The network platform is from the national/provincial/local environmental sector, and can actively real-time monitor the emission behavior of the key polluting industries (not open to public); The industries (only large ones) have their own emission monitoring networks (selectively open to public); The National Monitoring Centre conducts spot-checks of the local industries (not exclusively the key pollution industries) and compares the emission data with the industry self-monitoring data, and then reports to the MEP(Ministry of Environmental Protection). ^$^ Department of Pollution Prevention and Control of MEP is in charge of this action; The six Environmental Protection Supervision Centres (more details see Table A5), here the North China Environmental Protection Supervision Centre, also conduct supervision in BTH region.

Since the preparation for the Olympic Games, the central government has engaged in relocating heavy industries from Beijing to its surroundings, mainly to Hebei. Faced with high energy consumption, high pollution emission, and exacerbating environmental degradation, in 2013, the central government launched a national call to decrease the production capacity and to transform and upgrade the equipment and production processes in Hebei and other provinces. The concern about public health impacts of high air pollution has accelerated industry restructuring in the BTH region. Figure 2 indicates the industrial restructuring enforcement procedure in Hebei Province.

The implementation of the industrial re-structuring followed a top-down approach from central government to local government. The central government launched the general legislations on the emission standards (including technical standards) and plans on emission reduction, the provincial government set up the targets on the emission control and assigned the implementation tasks to local governments, and the local governments set up more specific plans and assigned specific implementation tasks to local industries (ID-08). In parallel, the local governments conducted routine monitoring and assessment of industrial emissions (including technical innovation) through three major pathways: routine emission monitoring, emission monitoring platform and public reporting involving third parties such as NGOs. The government monitoring platform was merely for internal evaluation, and for the industrial self-emission-monitoring system, the emission data can be made selectively accessible to the public depending on the consent of the respective industry (ID-08). For the industries that did not meet the emission standards, the government tried to enforce the improvement of emission standards mainly by confronting local administrators and industry representatives personally and/or suspending licenses and applying heavy fines. If the industrial emissions would still not meet the standards in due time, the government forced them to close down permanently. This applied especially to fragmented low-technology and high emission industries (ID-01). Social issues, mainly unemployment, emerged from closing down a significant number of industries as there was a lack of immediate alternative employment opportunities for often low skilled labour (ID-11). The process to solve the social issues attributable to industrial restructuring also followed a top-down pathway, through specific plans, social security support, financial support, and prioritized employment policies (Figure 2).

With the analysis of the interviews, we intended to explore the expert’s view on the following issues: (a) opportunities and challenges during the implementation of industrial restructuring; (b) the potential consequences including regional relationship in terms of air pollution control, and unintended social consequences that emerged from the industrial restructuring; and (c) the middle and long term sustainability in terms of industrial emissions reduction in BTH region.

##### Opportunities and Challenges for Implementation

The “*fast*” and “*decisive*” implementation of industrial restructuring showed the government’s resolve to control air pollution, but this short-term success on air pollution control could also be partially attributed to the decline in demand for steel (or overcapacity in the global market), as one interviewee stated. On the one hand, the government could take this opportunity to upgrade the technology and raise emission standards, but on the other hand, it had to be cautious for potential future increases in emissions if the international demand for steel should recover.

Challenges that are posing difficulties to the full enforcement of the emission control measures largely lay in the management and supervision of the process. There are still companies violating pollution emissions standards, which may be partly due to the “selective action” from local government. When it came to large state-owned companies, the authority and capacity of a local government were limited and it was hard to fully implement laws and policy measures [29]. Furthermore, some industries evaded routine government inspections; for example, industrial units that operated just during the night or were located in more remote rural areas, were often energy-intensive and had very high emissions. Suggestions by interviewed experts for improving implementation included enhancing the enforcement of industrial restructuring and stepping up monitoring. Further it was considered to be of importance to explore the relevant barriers to industry restructuring, for example the industries’ technical and financial capacity to upgrade in order to meet emission standards and to provide financial or policy incentives for industries to comply. Using economic measures such as developing an emission trading systems instead of merely command and control regulations, were also regarded to be helpful to push forward emission control.

##### Potential Regional Contradiction

It became obvious that regional contradiction could be caused by the relocation of industries due to the more stringent environment emission standards enforced in certain regions. As Beijing upgraded its industry, the relocation of less technologically advanced production facilities to neighbouring regions brought job opportunities and economic growth in these peripheral areas but also led to more pollution. The disparity in interviewees’ views regarding the impact of industry relocation stemmed from the different emphasis they placed on economic and environmental achievements. From an economic growth perspective, high-energy consumption industries can bring more employment and tax income to the Hebei province, which was economically favourable for the whole region. But from an environmental perspective, the economic development in BTH region, particularly in Beijing, was at the expense of environmental quality in Hebei [30], and the top-down enforcement on air pollution emission control, described as ”*political mission*” (Table 1, quote #13, #15), largely restricted the negotiation options between Beijing and Hebei, thus exacerbating the dissatisfaction in Hebei province. Either driven by the economic growth and/or by the political mission, those industries would bring extra pressure on air pollution emission control to the local government in the long term. Suggestions were to adopt a more effective platform for negotiation, to embed eco-environmental carrying capacity in the requirements for relocating industries and to provide “*financial subsidies, specific policies on market access and government procurement*” (Table 1, quote #18).

##### Social Problems

Industrial restructuring has generated serious impacts on employment. It was estimated that industrial restructuring could directly lead to more than 1,000,000 job losses by 2017 in Hebei Province [31]. Although the State Council launched “Guiding Opinions on Solving the Contradictions from Serious Overproduction” in 2013 to tackle the social problems, and followed by the Hebei provincial “Action Plans on Solving the Contradictions from Serious Over production” in 2014, unemployment is still a major social problem. Recent researched showed that, in Xingtai—a city in Hebei Province with 40% of local GDP from heavy polluting industries, 36.7% of the unemployment people laid off because of the industrial restructuring still could not find new job after the government resettlement plan [32]. Most of the unemployed were from small industries with less competitiveness in the job market because they were often “low-educated, low-skilled, and not insured”. This made their resettlement or re-employment even more difficult. Apart from unemployment, social inequity was another issue that the government was facing, as one interviewee remarked, “*Closing down the small and supporting the large industries, [but] who is paying the bill?*” (Table 1, quote #22). Several interviewees maintained that Beijing government should provide substantial financial subsidies to Hebei province and particularly specific policies on market access in Hebei Province, as the economic and industrial synergy support from Beijing to its nearby provinces was very limited [8]. This situation was enhanced since mid-2017, where, in order to meet the Action Plan target, the MEP released the “Action Plan to Comprehensive Control Autumn and Winter Air Pollution in Beijing-Tianjin-Hebei and Surrounding Regions 2017–2018” [33], and set up a supervision group of 1400 staff doing in-turn rigid accountability inspection in BTH, Shandong Province, Shanxi Province and Henan Province. This scheme significantly controlled the pollution emission, which is reflected in Figure 1, as the PM concentration and NO_2_ concentration in autumn and winter 2017 show remarkably low comparing with the former years, but potentially aggregated social issues.

##### Sustainability

Forced closing down of industries which are operating under legitimate business licenses would—from a legal point of view—require a “*reasonable compensation*” to those industries (Table 1, quote #24, #25). From a pollution emission perspective, as the technology and emission standards were updated, it was sustainable (Table 1, quote #26). However, in the long term, if regional dissatisfaction, social costs and legality issues cannot be alleviated, the full implementation of related air pollution control strategies would be less effective.

#### 3.2.3. Traffic Emission Control

Restrictions on vehicle registration, higher emissions standards and promoting clean energy vehicles are currently the three major approaches to decrease traffic emission in the BTH region. These are listed in Table A4.

##### Challenges for Implementation

According to the interviews (Table 2), the implementation of traffic emissions control was generally effective. Major challenges during the implementation were related to the comprehensiveness of identifying and controlling trucks and service vehicles that exceed emission standards, particularly at night and in rural areas. Further, some filling stations continued to provide low quality petrol or diesel. The interviewees’ suggestions to optimize control of road transport emissions can be summarized into three groups: further control to increase coverage of the total number of vehicles, promote active and public travel options (cycling, walking and public transport), and use environmental economic measures instead of command-control measures. Although the share of public and active transport in daily travel has been increasing, there remains much more scope to further increase its share, for example, by optimizing the allocation and exclusive reservation of routes to different modes of transport (e.g., bus or cycle routes) and improving comfort and convenience of public transport.

##### Sustainability

Several interviewees mentioned that vehicle restriction measures are violating personal freedom and property rights, and causing inconvenience for the general public, indicating that they were not sustainable as a long-term traffic control measures. Although phasing out old and yellow label vehicles (namely the gasoline vehicles that fail to meet the National I emission standard, and diesel vehicles that fail to meet the National III emission standard) was proceeding well in BTH, the way it was implemented was also arguably a violation of the individual’s property rights, for example, yellow label vehicles are forced to phase out even when those vehicles have not reached the end of the economic or technical life-time. Interviewees suggested alternative ways to phase out those type of old and yellow label vehicles including new-for-old trade or providing reasonable financial compensation.

#### 3.2.4. Collaboration on Air Pollution Control

There were regional collaborations with different focuses on air pollution control in BTH, the names and the functions see Table A5. The mechanism on regional collaboration to control air pollution was already well-established in the way of working groups, platforms and committees. The Olympic Games experience indicated that air quality improved dramatically with the short-term combined control measures, but the effects disappeared gradually afterwards [10]. According to our interviews, the greatest concern regarding long-term regional collaboration lies in how to ensure the adequate balance of interests among different local administrations. For example: Beijing, Tianjin and Hebei set up their own respective action plans according to their own interests as response to the economic growth and air pollution control pressure. This led to the decoupling of collaboration between regions when it came to the respective economic or environmental interests. Prioritized interests during implementation made it difficult to communicate among the three regions; for example “*save Beijing*” in the special events like APEC or hazardous pollution days (Table 3, quote #1). The three regional authorities are administratively at the same government level (provincial level), but Beijing, as capital and at the heart of the political centre, had more political clout and always received more attention nationally and internationally. An option suggested by the interviewees was to set up a judicially independent institution or coordinator with long-term authoritative strategy on air pollution control in the BTH region to ensure regional coherence and sustainable collaboration in the long run (Table 3, quote #4).

For the institutional collaboration, a decoupled relationship still existed between the policy makers and the technical advisors, mainly researchers, and between policy makers and the public, as one interviewee indicated: *“(although) many studies on the air pollution and health have been carried out; there is disconnection between government and research, and the public have limited knowledge of the air quality control strategies and health impacts. I suggest enhancing the public awareness on air pollution and health, pollution control plans, progress, etc. The related policy officers can participate in the research report and academic meetings to extend their knowledge. I also suggest regular training on related research for the government officers”* (ID-05).

## 4. Discussion and Recommendations

We summarized the progress on air pollution control in BTH, and explored the challenges for integrated air pollution control strategies in the region, focusing on industrial and traffic emission control measures. The measures used to control industrial emissions (mainly through industrial restructuring) and road traffic emissions have stopped the negative trend, and resulted in air quality improvements in the region. The air quality in BTH meets the targets from the Action Plan. The current Action Plan has specific focus on PM_2.5_ control; while NO_2_ showed slight decline and O_3_ could potentially increase, similar as Anger indicated [34]. A precaution strategy should be made to further control the multiple pollutants for the next air pollution control stage. One thing should be kept in mind is that, the Action Plan has been in place for 5 years and the result from a 5-year dataset might be less representative. These observed reductions are for sure related to the reductions in emissions, but also related to the meteorological conditions as interviewee indicated that the combination of specific climate and topography are influencing the formation and diffusion of air pollution [27,35]. Zou also revealed that the ice cap melting in the arctic contributed to the extreme poor ventilation conditions in China, which was related to the haze event in 2013 [36].

From a policy perspective, Zhang indicated that air pollution control during the Olympic Games of 2008 did not result in long term improvement due to the short-term nature of most policies implemented at that time [10]. However, under the current circumstances, the central government seems determined to control and reduce the air pollution, and has shown commitment to further engage on climate protection [6]. The continuity and consistency of air pollution control strategies are encouraging and a cause for cautious optimism. Still, the economic development and industrial production (mainly iron and steel industry in BTH) plays a crucial role in terms of air pollution control performance. With regard to the industrial restructuring, the local governments’ incapacity to enforce regulations, and the illegal emissions from certain polluters, were the major challenges during the implementation of the air quality plans. The weak institutional capacities of local government, when dealing with large companies and/or tax contributors hindered the full enforcement of the measures [8], and thus potentially increased social inequity, as small industries were forced to shut down. In addition, the air pollution control measures were introduced suddenly with a “command and control” approach, and the regional market in Hebei was far from being ready to absorb the redundant labour force [37]. Even with the policy and financial support from national to local government, unemployment is still the major social consequence of industrial restructuring in the Hebei province.

The sustainability of the air quality strategies based on industrial restructuring is debatable, mainly because of the top-down, command and control approach, and the insufficient compensation for the huge social cost (unemployment). Unlike PRD and YRD having better market-regulated economic development mechanisms, the economic development in BTH region is strongly influenced by the political enforcement [38]. Consequently, Beijing has very limited influence on the economic development of its neighbouring areas such as Hebei [29,38,39]. As the economic and environmental development in the region, particularly in Beijing, was at the expense of Hebei’s environmental quality [30], financial subsidy and substantial market support at central-local and cross-regional level, would be needed to reduce unemployment and mitigate the potential regional dissatisfaction in the long run [39].

In terms of traffic emission control, the interviews indicated that the related actions were effective, while the sustainability—at least in the long run—was questionable. Some of the actions were violating the individual’s property right. Individual resisted by covering parts of the license plates or borrowing license plates from other car owners thus undermining traffic restriction policies and thus the effectiveness on pollution emissions control [40]. Although these measures, particularly for vehicle license-plate lottery, limited the total vehicle numbers, and contribute to the air quality improvement, this kind of command and control rationing measures have a small immediate effect on traffic control and emissions, but the effect was short-lived and provided loopholes to circumvent the policy.

Few collaboration mechanisms were already in place, but the regional collaboration on air pollution control in BTH mainly focused on short-term control (emergency response or special events like Asia-Pacific Economic Cooperation (APEC) summit). Liu indicated that the bottleneck of regional collaboration lies in the administrative management system [30]. For this Beijing played a more active role while Hebei and Tianjin played more passive role in the collaboration. The quote “*save Beijing*” (Table 3, quote #1) indicates the contradiction between the central government which develops the broad policies and the local government which was responsible for implementing the policies. A long-term regional collaboration involving BTH and the surrounding regions is however indispensable, as the surrounding regions also contribute a large proportion (28–36%) of the pollutants observed in BTH [41,42].

The challenges for the success of long-term emission control mainly lie in the appropriate design of strategies themselves and in the way they were enforced, i.e., the overall governance of the strategies [43]. Although the government formulated laws and regulations on “public supervision mechanism” to promote transparency (Figure 2), public participation had a very limited role in reality [44,45]. A more substantive participation of the public and the third sector (e.g., NGOs) can facilitate the implementation of the measures. A transparent supervision mechanism in BTH can prevent “selective action” during the industrial restructuring, and transparent information disclosure mechanisms on industrial emissions from both industry and government can facilitate regional information sharing, enhance accountability, and increase public participation in both decision making and supervision [46,47,48]. In addition, although there are sufficient environmental legislations, this study indicates that a legal basis for the implementation and enforcement of the emission control measures is still wanting, even though this is -short termly-overcome by the in-turn rigid accountability inspection since mid-2017. This has major consequences for the sustainability of the strategies.

Apart from the lack of appropriate governance mechanisms, the pre-dominant emphasis on GDP growth, and still subordinated and limited attention on environmental protection in the government’s performance evaluation encourages the local officials to focus on economic growth, rather than environmental protection, as their priority [27].

## 5. Conclusions

Using both quantitative and qualitative methods, we explored the progress and challenges of the air pollution control in the BTH region in terms of industrial and traffic emissions. In general, air quality in the BTH region can meet the 5 years target of Air Pollution Prevention and Control Action Plan. The implementation of both industrial restructuring and traffic emission control has provided environmental benefits, but more stringent monitoring and supervision is needed to improve enforcement. Social injustice and unemployment were the main unintended consequences of the policy that require attention from national, regional and local authorities. In the long term, the mandatory top down enforcement with little self-regulation mechanism could make the strategies unsustainable. Current collaboration on air pollution control mainly focuses on emergency haze episode control, but not on routine long-term air pollution control. Better mechanism to provide a multi-sectoral communication between regions is still lacking. The interview results revealed the challenges that can hinder the effectiveness and sustainability of the air pollution control plans, and also shed light on the lack of appropriate governance mechanism in terms of decision-making and implementation. More active stakeholder involvement could help mitigate unintended consequences like social injustice, unemployment, and regional contradiction. Further, it could improve the intrinsic motivation for the local government to implement the measures, to increase the implementation and monitoring, and thus increase the sustainability of the strategies. A sound information exchange system for decision-making, policy implementation and monitoring, would improve supervision and increase the accountability. Finally, strengthening the legal basis for emission trading, compensation mechanisms and enforcement in the region and more importantly between regions, can improve consistency and fairness of policies, and ensure the interests can be reasonably balanced between regions.

## Figures and Tables

**Table 1:** Interview quotes on industrial restructuring and related emission control.

Nr.	Items	The Quote	Quote ID
**Progress in Implementation**			
1	Progress	*“Close, suspend, or restructuring are the main themes to control industrial emissions”*	(ID-01, ID-03)
2		*“Implementation enforcement is very fast and the target is very clear”*	(ID-03)
3		*“According to our investigation, the local government just either closes the high polluting industries or suspends them until they meet the emission standards within a certain time period. It is very decisive”*	(ID-01)
4		*“the international overcapacity in the steel industry in the past few years provided a very good opportunity for the industrial re-structuring in BTH region”*	(ID-05)
5	Problem	*“Monitoring and supervision issues remain as emissions are still exceeding the standards”*	(ID-01, ID-03)
6		*“The government management pattern for industrial emissions has shifted from inaction to selective action”, and “most of the closed or suspended industries are small-sized and inferior”*	(ID-01, ID-10)
7		*“Personally, I think the ‘marginal effect’ of the policy is decreasing, … some local governments focus more on economic development and government performance, verbally paying attention on the industrial emission control, but ignoring it in practice. They pay attention formally, but not in practice, and it is difficult to tell how serious it is. Some industries run during the night and close during the daytime”*	(ID-04)
8	Suggestion	*“…we also should study industries’ behaviour to explore the reasons for the excessive emissions; whether they (industries) do not care about environment protection or they just cannot afford to upgrade their installations”; “we also need to consider their emissions reduction capacity…”*	(ID-03,10)
9		*“More research on the pollution source, the diffusion, and the interaction mechanism needs to be done to support policy”*	(ID-03)
10		*“Improve the emission’s charging system, and establish an emissions trading policy”*	(ID-12)
11		*“Enhance the monitoring, law, punishment…”*	(ID-01, ID-02, ID-03)
12		*“Provide incentive strategies to promote restructuring”*	(ID-03)
**Potential for Regional Contradiction**			
13	Not necessarily	*“The relocation will inevitably lead to the emission and pollution relocation, but not necessarily lead to regional contradiction as the local government (HB) is willing to welcome industries that can generate GDP growth. The relocation is a top-down political mission from the higher government, a kind of a mandatory mission”*	(ID-02, ID-08)
14	Yes	*“Yes, it will cause regional contradiction”*	(ID-06)
15		*“Current action on air pollution control is more a political mission but not enforced by law. In the long run, there will be inevitable contradictions of interest between regions”*	(ID-09)
16	Suggestion	*“…the central government should provide mechanism to settle the contradiction, such as set up regular meetings among the regions…”*	(ID-09)
17		*“Embedding environmental carrying capacity, when re-locating industries (to Hebei)”*	(ID-07)
18		*“Increase the environmental protection and energy consumption standards, promote recycling economy and clean production; in the meantime, Beijing should increase support to control pollution in Hebei and Tianjin, including increase financial subsidies, specific policies on market access and government procurement, etc.”*	(ID-06)
**Social Problems**			
19	*“The restructuring (mainly closing the small industries) impacts the local job market seriously”*		(ID-11)
20	*“The control target and the economic growth sometimes is not balanced among regions, and we can see that the social cost is huge”*		(ID-02)
21	*“Many people are unemployed; while formal employee have some insurance, the situation is harsh for temporary employees (most are in the small industries), who normally are low-educated, low-skilled, and not insured“*		(ID-01, ID-03, ID-11)
22	*“Closing down the small and supporting the large industries, [but] who is paying the bill? How to balance the interests between different population groups? Should it be considered during the drafting, implementing and assessment of air pollution control actions? ”*		(ID-11)
23	*“How to improve social security (for workers laid off due to restructuring)?”*		(ID-02, ID-03, ID-11)
**Sustainability**			
24	*“Obliged suspending or closing of industries, for short period during an (air quality) emergency or special event, is acceptable, but not as a normal or permanent action unless there is reasonable compensation”*		([ID-03)
25	*“A reasonable compensation among the regions should be provided; monitoring of the compensation needs to be introduced to make sure the money is used in pollution control/industrial restructuring related things”*		(ID-09)
26	*“It is fairly sustainable, because the guidelines, emission standards, industrial technique have already improved to certain level; if the monitoring and supervision continues to be strict, it is unlikely to return to the pervious problems”*		(ID-05)
27	*“… particularly when the steel market is recovering, supervision and inspection will inevitably become more difficult, and the likelihood for excessive emissions will increase. To maintain the sustainability of the action plan, it is imperative to step up penalties, and normalize and popularize the reporting mechanism”*		(ID-04)
28	*“Related laws and regulations need to be fully implemented to ensure sustainability, such as enhancing the monitoring of fuel quality, building up a third party-monitoring institution, empowering environmental agencies and clarifying their rights; more emphasis has to be give on environment protection in the government performance evaluation”*		(ID-12)

**Table 2:** Interview quotes on traffic emission control.

Nr.	Items	The Quote	Quote ID
**General Opinions**			
1	Challenge	*“Most of the transportation emission control measures are going relatively well”*	(ID-02, ID-03)
2		*“Diesel and gasoline quality varies in different gas stations, some of which still do not meet the diesel and gasoline standards.”*	(ID-02)
3		*“Vehicle emissions in rural area are still high because vehicles do not reach the emissions standard”*	(ID-01, ID-03)
4		*“The vehicles, particularly trucks, which are only limited to enter BJ/TJ at night, do not meet the emission standard”*	(ID-01)
5	Suggestion	*“Further control the total vehicle number”*	(ID-03)
6		*“Further improve diesel and gasoline quality and vehicle emissions standard”*	(ID-02, ID-03)
7		*“Optimize the allocation and the use of the public transport, and also improve the comfort of public transit”*	(ID-03, ID-04)
8		*“Build up bicycle paths in some areas and promote green travel”*	(ID-02)
9		*“I do not recommend compulsory emission control actions like traffic restrictions, but go for economic measures like increasing parking fees and oil prices, etc.”*	(ID-04)
10		*“We could use environment economic measures to regulate travel behaviour … Which is affecting the pollution? The emission factor, mileage and the number of the vehicles … for example, price measures can obviously change the mileage, emissions and short-distance travel patterns … at the early stage of pollution control, the net benefit of these kinds of command and compulsory policies are significant, but with the improvement of the pollution control targets, behavior change induced by environmental economic are more manifest”*	(ID-03)
**Sustainability**			
11	*“[but] if the vehicle restriction becomes normal, for example, for a half year or one year, it obviously goes beyond the individual property rights according to the law”*		(ID-02, ID-09)
12	*“As we all know, there is no time limit (except for mileage) to retire private cars, so if you want to phase out the old cars, you can only encourage people to do so through for example new for old trade or providing subsidies, but not saying the car has to be retired after 10 years, this is inappropriate”*		(ID-09)
13	*“The traffic emission control actions (traffic restriction) contribute to the decrease of the air pollutants, like military parade blue and APEC blue, but we cannot deny that even with the traffic restriction, the heavy smog still exists. We should also notice the side effect of the traffic control, which is the inconvenience for the public, it is not sustainable and should not be used as a routine measure to control air pollution”*		(ID-04)
14	*“Traffic restrictions and license lottery cannot reasonably decrease the total number of vehicles, which in the meanwhile, impose dissatisfaction from the public. It is not sustainable and I suggest to take economic measures like parking tax”*		(ID-02, ID-03, ID-04)

**Table 3:** Interview quotes in regional and institutional collaboration.

Nr.	The Progress, Challenges and Suggestions	Quote ID
1	*“There is regional collaboration, but still not close enough; the air pollution control measures in BTH are always ‘prosperity or loss’; it is necessary to build up a tight collaboration with the collaboration mechanism more mature; and now in most of the cases, the surrounding regions together ‘save Beijing’”*	(ID-04)
2	*“The regional collaboration is not smooth, each government cares about its own interests and constituency, and it is very difficult to communicate among the institutions involved.”*	(ID-08)
3	“*Very limited collaboration with the other surrounding, like Shandong, Shanxi province, but it is needed; for BTH, the conventional collaboration. The BTH collaboration is more for the special events or emergency but not for regular collaboration, which needs to be enhanced. Beijing, Tianjin and Hebei are three politically parallel governments, it is still lack of coordination mechanisms and a political institution or organization which is separate from or above the three to coordinate the implementation of the action plan in BTH*”	(ID-03)
4	*“Suggest to set up a regional environmental centre (commission) for joint prevention and control (also ID-03,* *ID-06), and empower independent right (with judicial independence), and with fixed fund source and independent right to allocate the funds and subsidies (for example, certain percentage of GDP from each province/city), and have the right of carrying out punishment and one-vote veto, and etc.”*	(ID-12)
5	“For the three major organizations * recently set up for regional air pollution prevention and control, we need to be keep in mind that, if they are temporary organizations, then the authority and stability are questionable, and the lack of continuity of the policy can also influence the authority and stability”	(ID-09)
**Regional Emergency Response System**		
6	*“The emergency system is already in place, and can provide timely information, but the corresponding measurements are mainly limited to temporarily pause the production and vehicle restriction, both are not a sustainable measures”*	(ID-03, ID-04)
	*“… but the forecast is too late, the measures can only be taken in the same day when heavy pollution happens, thus the measures cannot decrease the pollution until a while. The prediction timeliness should be improved to provide ample time for preparation and reaction”*	(ID-06)

* the three major organizations here mean the Working group office air pollution prevention and control of BTH region and its surrounding, Air pollution early warning and forecast platform and Coordination group office of vehicle emission control in BTH and its surrounding. Their functions can be found in Table A5.

## Appendix B. The Calculation Method and Classification of IAQI and AQI

Technical Regulation on Ambient Air Quality Index (HJ 633-2012) and Ambient Air Quality Standards (GB3095-2012) defined the calculation and classification of the IAQI and AQI. The AQI is calculated through Individual Air Quality Index (IAQI), and the calculation base for IAQI and AQI is explained as following.

**Table A1:** The corresponding concentration limit value for individual air quality index (IAQI).

IAQI	Concentration Limit Value									
	SO_2_-24 h µg/m^3^	SO_2_-1 h(1) µg/m^3^	NO_2_-24 h µg/m^3^	NO_2_-1 h(1) µg/m^3^	PM_10_-24 h µg/m^3^	CO-24 h mg/m^3^	CO-1 h(1) mg/m^3^	O_3_-1 h µg/m^3^	O_3_-8 h µg/m^3^	PM_2.5_-24 h µg/m^3^
0	0	0	0	0	0	0	0	0	0	0
50	50	150	40	100	50	2	5	160	100	35
100	150	500	80	200	150	4	10	200	160	75
150	475	650	180	700	250	14	35	300	215	115
200	800	800	280	1200	350	24	60	400	265	150
300	1600	(2)	565	2340	420	36	90	800	800	250
400	2100	(2)	750	3090	500	48	120	1000	(3)	350
500	2620	(2)	940	3840	600	60	150	1200	(3)	500

Notes: (1) 1 h average concentrations of SO_2_, NO_2_ and CO are used merely for real time report. 24 h average concentrations are used for the daily air pollution report; (2) When the 1 h average SO_2_ concentration is higher than 800 μg/m^3^, it is not used for IAQI calculation anymore. Only 24 h average SO_2_ concentration is used for IAQI calculation; (3) When the 8 h average O_3_ concentration is higher than 800 μg/m^3^, it is not used for IAQI calculation anymore. Only 1 h average O_3_ concentration is used for IAQI calculation.

Then, the *IAQI* is calculated by the following formula:

(A1) $${\mathrm{IAQI}}_{P}=\frac{IAQI_{hi}-IAQI_{lo}}{BP_{hi}-BP_{lo}}\left(C_{p}-BP_{lo}\right)+IAQI_{lo}$$

where, ${\mathrm{IAQI}}_{P}$ is the *IAQI* for pollutant *p*; $C_{p}$ is the concentration of pollutant *p*; $BP_{hi}$ is the high limit value for $C_{p}$ according to Table A1; $BP_{lo}$ is the low limit value for $C_{p}$ in Table A1; $IAQI_{hi}$ is the corresponding *IAQI* for $BP_{hi}$; and $IAQI_{lo}$ is the corresponding *IAQI* for $BP_{lo}$.

In the end, the *AQI* is calculated by the following formula:

(A2) $$AQI=\max\left\{IAQI_{1},\;IAQI_{2}\ldots IAQI_{n}\right\}$$

where, *AQI* is the air quality index, *n* is the number of the included pollutants. When *AQI* is higher than 50, pollutant has the largest *IAQI* is the dominant pollutant (can be two or more dominant pollutants). When IAQI is higher than 100, it is the non-attainment pollutant.

The level of *AQI* is classified as following: 0–50, level I, describes as excellent quality; 51–100, level II, describes as good quality; 101–150, level III, describes as mildly polluted; 151–200, level IV, describes as moderately polluted; 201–300, level V, describes as heavily polluted; >300, level VI, describes as severely polluted.

## Appendix C. Interview Guidelines

**Overall aim of the interview**: to explore the stakeholder’s view on the ‘air pollution prevention and control action plan’ in Beijing-Tianjin-Hebei region (BTH).

**Expected Outcomes**

(1) The challenges and experiences during the implementation of the action plan (collaboration, interests conflict, management and others);
(2) Apart from existing actions, standards, measurement, and laws, what else can be further done to control air pollution.

**Interview Guide**

1. Personal context;
2. Have you been involved in any of the strategies on air pollution control, in the way of drafting, researching, propagating, implementing, evaluating, monitoring, or some other way? (in another way, if you familiar with the action plan).

❖ **General Question**
  1. What do you think of the ‘air pollution prevention and control action plan’ (progress, the challenges, sustainability and further suggestions)?
❖ **Industrial Relocation/Restructuring**
  2. What are the major challenges during restructuring? Do you think the restructuring has led to the expected emission control?
  3. According to the report, the industrial-restructuring is going well, particularly in Hebei province, and the air quality improved, but for some regions, the air pollution is still far from reaching the target, what do you think the reasons are?
  4. Do you think industrial relocation in the Integrated-BTH will add additional burden to Tianjin and Hebei on air pollution control, and thus lead to conflict between BJ and T-H? Why and how to alleviate the burden for Tianjin and Hebei?
  5. Regarding the industrial emission reduction, do you suggest further control? What kinds of measures do you suggest?
❖ **Traffic Emission Control**
  6. How do you evaluate of the current transportation emission control measures? (progress, the challenges, sustainability)
  7. Do you suggest further transportation control? What kinds of measures do you suggest?
❖ **Regional and Institutional Collaboration**
  8. What do you think of the current collaboration on air pollution control in Beijing-Tianjin-Hebei? What are the challenges and what do you suggest to improve?
  9. Is there any close collaboration between BTH and other surrounding regions, like Shandong province, Shanxi province, and Inner Mongolia in terms of air pollution control? If not, do you think it is necessary, and in which way?
  10. Based on your work/research, can you give some suggestions on air pollution control for the next “five year plan” (13th-five year plan)
  11. Are the heavy pollution weather monitoring and warning system in place? Can they provide timely and useful information for emergency response?

## Appendix D

**Table A2:** Major air quality control strategies since 2012.

Measures	Issued by *	Issue/Execute Date	Type
12th Five-Year Plan	State council	2011–2015	Plan
13th Five-Year Plan	State council	2016–2020	Plan
Environmental protection law	NPC	Amended 2014.04	Law
Air pollution prevention and control law	NPC	Passed 2014.04 Executed 2016.01	Law
Clean Production Promotion law	NPC	Executed 2012.07	Law
The 12th FYP on Prevention and Control of Air Pollution in Key Regions	MEP, NDFC, MoF	Issued 2012.10	Plan
Air pollution prevention and control action plan	State Council	Issued 2013.09	Plan
National 10 measures	State Council	Published 2013.06	Plan attachment
Performance Assessment Measures for Air Pollution Prevention and Control Action Plan	State Council	Issued 2014.07	Plan attachment
National Ambient Air Quality Standard	MEP	Executed 2016.01	Standard
Action Plan to Comprehensive Control Autumn and Winter Air Pollution in Beijing-Tianjin-Hebei and Surrounding Regions 2017–2018	MEP	Executed 2017.08	Plan
Emission standard of air pollutants for industries ^#^	MEP, AQSIQ	Varied	Standard
Limits and measurement methods for emissions from light-duty vehicles (V)	MEP, AQSIQ	Issued in 2013.09 Fully executed 2018.08	Standard
Gasoline for motor vehicles Automobile diesel fuels (V)	AQSIQ, SA	Executed 2013.12/06	Standard
Rules on the standard for compulsory retirement of motor vehicles	MoC, NDFC, MoPS, MEP	Executed 2013.05	Standard

* Abbreviations: NPC, National People’s Congress; MEP, Ministry of Environmental Protection of the People’s Republic of China; MoF, Ministry of Finance; NDFC, National Development and Reform Commission; MoPS, Ministry of Public Security; Ministry of Commerce; AQSIQ, General Administration of Quality Supervision, Inspection and Quarantine; SA, Standardization Administration. ^#^ mainly includes the cement industry, iron and steel industries, brick and tile industry, electronic glass industry, iron smelting industry steel rolling industry, etc.

**Table A3:** Particulate matter decrease targets for the Action Plan at provincial or municipal level.

Province	PM_2.5_ Decrease by *	Province	PM_10_ Decrease by **	Province	PM_10_ Decrease by
Beijing	−25%/limit to 60 μg/m^3^	Henan	−15%	Sichuan	−10%
Tianjin	−25%	Shaanxi	−15%	Ningxia	−10%
Hebei	−25%	Qinghai	−15%	Heilongjiang	−5%
Shanxi	−20%	Xinjiang	−15%	Fujian	−5%
Shanghai	−20%	Hubei	−12%	Jiangxi	−5%
Jiangsu	−20%	Gansu	−12%	Guangxi	−5%
Zhejiang	−20%	Liaoning	−10%	Guizhou	−5%
Shandong	−20%	Jilin	−10%	Hainan	Continuously decrease
Guangdong	−15% PRD *	Anhui	−10%	Yunnan	Continuously decrease
Chongqing	−15%	Hunan	−10%	Tibet	Continuously decrease
Inner Mongolia	−10%	Guangdong	−10%		

* For PM_2.5_, the baseline is the provincial/municipal annual PM_2.5_ concentration in 2013, when the Ambient air quality standards (GB 3095-2012) were first introduced in China; ** For PM_10_, the baseline is the provincial/municipal adjusted annual PM_10_ concentration in 2012 (GB 3095-1996).

**Table A4:** Major traffic related emission control measures in BTH ^#^.

Measures	Region and Description
Improve emission standards for vehicles	(BTH) National V emission standards (same as EU standards) for all vehicles
Improve gasoline and diesel quality	(BTH) Improve gasoline and diesel quality to meet the National V emission standards
License-plate lottery	(BJ, TJ) Limiting the number of new license plates per year, to reduce the registration of additional cars
Vehicle restriction rule	BJ, TJ and some cities in HB restrict the use of private cars for one work-day per week according to the last number of the license plate, or during rush hour; for specific events or on extremely polluted days, alternatively odd or even license plates only are allowed to enter urban areas. This is also aimed to alleviate congestion problem
Promoting clean energy and new energy vehicles	(BTH) The government(Ministry of Finance) provides financial incentives for buying clean-energy vehicles; provides tax discounts for purchasing clean-energy vehicles; builds up charging facilities for electric vehicles; exempts clean energy vehicles from the lottery for license plates; and increases the annual quota * for clean energy vehicles in BJ and TJ
Phasing out old and yellow label vehicles **	(BTH) Provide financial subsidies for phasing out yellow label vehicles which have not reached the retire years; yellow label vehicles are banned from entering urban core areas

^#^ BTH, Beijing-Tianjin-Hebei; BJ-Beijing, TJ-Tianjin, HB-Hebei; * in order to control the total vehicle number, local governments set up annual ceilings for the total amount of vehicles, and control the vehicle number through license plates; ** yellow label vehicles, namely the gasoline vehicles that fail to meet the National I emission standard (the same with corresponding Euro I emission standard), and diesel vehicles that fail to meet the National III emission standard (the same with corresponded Euro III emission standard). Those vehicles are labelled with yellow environmental protection certificates.

**Table A5:** The major regional collaboration mechanisms on air pollution control in BTH region.

Name	Key Functions
Environmental protection supervision centre *	The main tasks for Environmental protection supervision centre are: to supervise the implementation of national environmental policies, plans, laws and other legislations; to investigate major environmental pollution incidents; to coordinate major environmental disputes between regions; to supervise the emergency response for major environmental incidents; to inspect the enforcement of environmental laws and sewage charges; to provide suggestion on limited batch for different regions and different industry types; to inspect the state-controlled emission sources ^#^; to inspect the environmental protection enforcement in environmental function area, national nature reserve area and national key ecological protection area; to undertake or coordinate the cross-provincial environmental pollution or ecological destruction cases.
Working group office air pollution prevention and control of BTH region and its surrounding	The working group includes the Ministry of Environmental Protection, the National Development and Reform Commission, the Ministry of Industry and Information Technology, the Ministry of Finance, the Ministry of Housing and Urban-Rural Development, the Meteorological Bureau, the Energy Bureau, and the Ministry of Transport. The working group makes the annual plans on emission decreasing tasks and implements the tasks; and makes the medium and long term planning on regional air pollution control.
Coordination group office of vehicle emission control in BTH and its surrounding	The functions include cross-regional punishment for vehicle excessive emission, regional vehicle emission data sharing, joint spot-check for new vehicles in BTH, Shanxi province, Inner Mongolia and Shandong province.
Experts committee on regional air pollution prevention and control	The main tasks are: to identify research focus on the air pollution prevention and control; to provide guidance on composing the air pollution prevention and control plans; conduct fundamental research on source appointment, regional transmission, provide suggestion on advanced and applicable air pollution emission control technology
Air pollution early warning and forecast platform	This platform is mainly for severely polluted episodes or special events. It can provide synchronous monitoring of the regional air quality and provide real-time video meeting within the BTH and its surrounding.
Information sharing platform for air pollution prevention in BTH and its surrounding	The platform is to provide air quality data and key polluted emission sources information sharing in BTH, Shanxi province, Inner Mongolia and Shandong province.

* There are six centres: Northern, Eastern, Southern, Northwest, Southwest and Northeast. The Northern Environmental protection supervision centre is in charge of the BTH region and its surrounding. Normally, the surrounding of BTH region in terms of air pollution control includes Shanxi province, Shandong province, Inner Mongolia, and Henan province. ^#^ Based on the amount and the type of the emissions; the Ministry of Environmental Protection classifies the emission sources into state-controlled, provincial-controlled and municipal-controlled.
